# Recurrent bacteremia as presentation from an aorto-duodenal fistula after endovascular aneurysm repair

**DOI:** 10.1016/j.idcr.2022.e01566

**Published:** 2022-07-13

**Authors:** Hsing-yun Lee, Yu-Cheng Tung, Chen-Hsiang Lee

**Affiliations:** a Department of Internal Medicine, Kaohsiung Chang Gung Memorial Hospital, Kaohsiung, Taiwan; b Department of Radiology, Kaohsiung Chang Gung Memorial Hospital, Kaohsiung, Taiwan; c Division of Infectious Diseases, Department of Internal Medicine, Kaohsiung Chang Gung Memorial Hospital, Kaohsiung, Taiwan; d Chang Gung University College of Medicine, Kaohsiung, Taiwan

**Keywords:** Aneurysm, Fistula, Bacteremia

An 83-year-old man with a history of stroke had a fever and back pain that persisted for 3 months as well as episode 3 years prior. Blood culture yielded *Escherichia coli*. Whole-body inflammation scan revealed inflammation in the abdominal para-aortic region. Abdominal computed tomography (CT) revealed a 1.9-cm infrarenal aortic saccular aneurysm, periaortic soft-tissue infiltration with rim aortic-wall enhancement, and a 5.2-cm retroperitoneal para-aortic hematoma compatible with abdominal aortic aneurysms (AAA) rupture. He received endovascular aortic repair (EVAR) immediately and was discharged with oral antibiotic treatment after 1 week of observation. However, he experienced multiple bacteremia episodes caused by different species of *Enterobacteriaceae* 2 years after EVAR. CT angiography of the aorta revealed no evidence of recurrent mycotic aneurysm or abscess formation but loss of the fat plane between the duodenum and aorta (Panel A, arrow). Compared with an image obtained 1 year prior, the duodenal wall appears adherent to the aorta but was preserved between the duodenum and aorta (Panel B, arrow). Anemia and gastrointestinal bleeding were treated endoscopically. A metallic foreign body was found in the third portion of the duodenum (Panel C). A post-EVAR aortoenteric fistula (AEF) was identified. Surgical repair was performed after sepsis was relieved. He recovered completely postoperatively.

EVAR significantly reduced the 30-day mortality when compared with open surgery [1]. However, patients who receive EVAR experience higher mortality than those who receive open surgery after 8 years of follow-up. The main reason for increased mortality is the secondary aneurysm sac rupture [2].

AEF is one of the most fatal complications associated with graft repair. Even when an operative intervention is performed, the mortality rate is as high as 50 % [3]. The risk factors for AEF include a history of receiving an aortic intervention, peptic ulcer, gastrointestinal malignancy, and para-aortic radiation. A possible mechanism of AEF is persistent inflammation. This causes degradation of the aortic graft anastomosis, resulting in pseudoaneurysm formation. Moreover, inflammation induces aortic adhesion to the bowel wall. Mass stress with pulsation movement of the bowel ultimately contributes to the AEF [3]. The most frequent location of AEF and bowel connection is the duodenum [4] which also occurred in our patient. The clinical presentation of AEF may be non-specific symptoms such as fever, hematochezia, back pain, or abdominal pain. Therefore, a history of duodenal ulcer, persistent aortic stent infection, and multiple episodes of *Enterobacteriaceae* bacteremia suggest that clinicians should be aware of AEF. (Fig. 1).

**Fig. 1:**
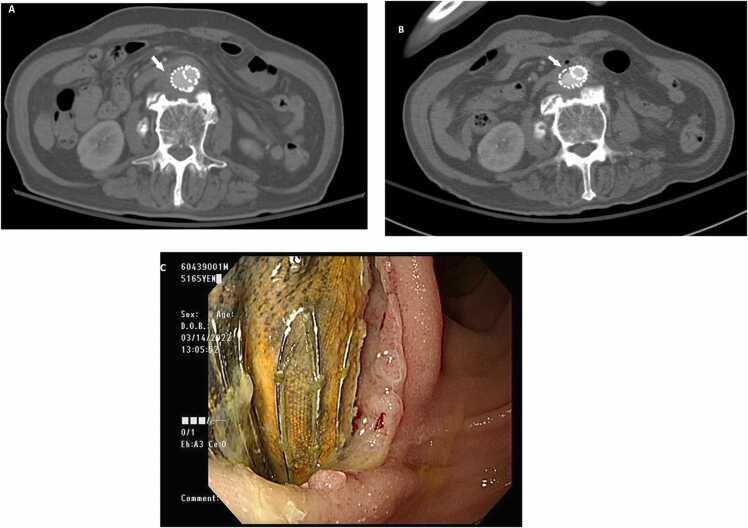
Plane A The duodenal wall was adherent to the aorta; however, the fat plane between the duodenum and aorta was preserved. Plane B Arrow site showed loss of the fat plane between duodenum and aorta. Plane C A metallic foreign body suspected of an aortic stent was found in the second portion of the duodenum.

## Ethical approval and Consent

Written informed consent was obtained from the patient for publication of this case report and accompanying images.

## Funding

This research did not receive any specific grant from funding agencies in the public, commercial, or not-for-profit sectors.

## CRediT authorship contribution statement

Hsing-yun Lee: Writing – original draft, Conceptualization. Yu-Cheng Tung: Writing – review & editing. Chen-Hsiang Lee: Writing – review & editing, Supervision. All authors contributed to the writing of the final manuscript.

## Conflicts of interest

The authors declare no conflict of interest.
